# Observations upon Sulphurous Mineral Waters

**Published:** 1808-08

**Authors:** M. Westrumb


*Observations upon Sulphurous Mineral Waters.*
By M. Westrumb.*

M. WESTRUMB has been employed in making researches upon several kinds of sulphurous waters, and latterly upon those of Eilsen in the county of Scaumbourg.

One of the most interesting facts he has discovered is, that all sulphurous waters contain a greater or less quantity of hydro-sulphuret of lime.

In order to ascertain it, he boiled the mineral water ex-

* From M. Gehlen's Journal of Chemistry, vol. v.

cluded from the contact of the air, in order to expel from it the sulphuretted hydrogen gas and the carbonic acid.

He afterwards poured into the residue—

1st, Sulphuric acid, which liberated sulphuretted hydrogen gas from it; and sulphat of lime was precipitated.

2d, Smoking nitric acid, which separated sulphur from it.

3d, Oxalic acid, which liberated sulphuretted hydrogen gas from it; and oxalate of lime was formed.

4th, The water evaporated with the contact of the air, precipitated sulphat of lime; and the sulphuretted hydrogen gas was liberated.

In order to determine rigorously the quantity of the sulphuretted hydrogen gas and of the carbonic acid, M. Westrumb proceeds in the following manner:—He introduces sulphurous water into a matrass to a certain point, putting a mark upon the level of the liquid; he adapts a curved tube to it, entering into a long cylinder, which is at one time filled with lime-water, and another time with acetate of lead, with excess of acetic acid.

The apparatus being thus arranged and well luted, he boils the water, and continues the ebullition until there is no more gas liberated.

In the first experiment it is the carbonat of lime which is precipitated, 20 grains of which correspond to ten cubic inches of carbonic acid; and in the second case it is oxidated hydro-sulphuret of lead, 19 grains of which indicate ten cubic inches of sulphuretted hydrogen gas.

Another observation equally remarkable concerns the sulphuretted hydrogen gas.

M. Gimbernat, a Spanish chemist, has asserted, that the hot springs of Aix la-Chapelle contains sulphuretted azotic gas.—M. Schaub attempted to extract it from the sulphurous waters of Nenndorf in Hesse. The following characters have been attributed to this gas:—1st, A smell similar to the sulphuretted hydrogen gas; 2d, Being indecomposable by the carbonic acid gas; 3d, Not being inflammable; 4th, Being improper for supporting an inflamed body; 5th, Being indecomposable by the nitrous gas; 6th, Being decomposed by the concentrated nitric acid, which separates sulphur from it; 7th, Decomposing the metallic solutions, and forming sulphurets; 8th, Having a great affinity for water, from which it cannot be separated, except by long ebullition.

But M. Westrumb has found that when we wash sulphuretted hydrogen gas with lime-water, or when we pass a current a current of this gas through slaked lime, it acquires all the above properties.

Whether the sulphuretted hydrogen gas be obtained from sulphurous waters, or be prepared in any other way in use, the same phænomena take place.

On bringing back the lime-water by an acid, sulphuretted hydrogen gas is liberated, which is inflammable, and which possesses its ordinary properties.

Sulphuretted azotic gas is therefore a product of the operation.

M. Westrumb does not yet know if this new gas be produced by the action of quick-lime upon sulphuretted hydrogen gas, or if this substance does not contain sulphuretted azote.

Lastly, a third observation not less interesting is the presence of carbon and carbonated substances in the sulphurous mineral waters.

M. Westrumb has discovered a new principle in them—a *fœtid resin of sulphur.* (Stinkendes schwefelharz.)

In order to obtain it, we must evaporate the sulphurous water excluded from the contact of the air, and afterwards take up the residue by alcohol, which dissolves this resin more than the earthy muriates. By the evaporation of the alcoholic liquid, the substance at first looks like a yellowish fat, it is successively coloured brown, and becomes resinous.

By repeated solutions in alcohol, and by evaporations, it is decomposed into sulphur, and into a resin of a blackish-brown.

It has a garlic smell, which becomes very strong, and similar to assafœtida, when we pour water into the alcoholic solution.

Its solution acts like an acid.

Its resin is dissolved in ammonia, and communicates a yellow colour to it; the liquor acts like that of Beguin. With lime-water we obtain a hydro-sulphuret. All these solutions act upon the metallic combinations like sulphuretted hydrogen.

As sulphurous mineral waters have their origin in beds of pit-coal, we might perhaps find the source of this bituminous principle in pit-coal itself.

Around the baths of Eilsen, like those of St Amand, there is accumulated a kind of crust, which gradually becomes of a dark colour, and latterly black.

By analysis, there have been extracted from it sulphuretted fetid resin, hydro-sulphuret of lime, sulphur, lime,

 alumine, alumine, magnesia, charcoal, and sand, with some fibrous substances, a little sulphuretted hydrogen gas, and carbonic acid gas.

Whatever be the origin of the bituminous principle in sulphurous waters, M. Westrumb succeeded in producing charcoal and fetid resin, by employing sulphur perfectly pure.

For this purpose he digested sulphur precipitated by an acid in alcohol. By distilling the alcoholic liquor, there is separated yellow crystalline sulphur, or a yellowish grey powder: the fetid resin is then completely formed in the liquor floating above, and possessing all the above properties.

We might attribute its formation to the presence of the alcohol, and *à fortiori,* because, after its separation from the residue of the evaporated sulphurous water, the penetrating smell is manifested, when it is taken up by the alcohol. But several observations lead M. Westrumb to think that the alcohol does not contribute to the formation, and that it rather derives its origin from the sulphur itself.

---

### *Letter of M.* Roloff, *of Magdebourg, upon the foregoing Subject.**

I have recently recognised, in an unexpected manner, the sulphuretted fetid resin of M. Westrumb.

M. Michaelis, after having precipitated the golden sulphat of the hydrogenated sulphuret of antimoniated potash, evaporated the liquor floating above containing the sulphat of potash.

When the ley began to concentrate, a vapour was developed, which very much embarrassed the artist who was stirring the mass. There was at the same time manifested an insupportable smell, analagous to that of burnt assafœtida.

The saline mass evaporated to dryness had a grey colour, and the remarkable smell we have mentioned.

It was was put in digestion with alcohol, which acquired the taste and smell of garlic.

The alcoholic liquor evaporated spontaneously, yielded a grey gluey mass, possessing the same smell and taste.

I am desirous that this experiment should be made public, not knowing if M. Westrumb is acquainted with the formation

* From Gehlen's new Journal of Chemistry.

formation of a large quantity of fetid resin which may be easily procured by this process.

As the smell is manifested before putting in the alcohol, we may conclude, with M. Westrumb, that the alcohol does not contribute to its formation.

